# To the Dentists of the United States

**Published:** 1895-07

**Authors:** 


### To the Dentists of the United States.

### CHAPIN A. HARRIS MEMORIAL.

A movement has been inaugurated in Baltimore which has received the earnest endorsement of Dr. M. W. Foster, Dean of the Baltimore College of Dental Surgery; Dr. F. J. S. Gorgas, Dean of the University of Maryland and other prominent members of the profession of that city. It is proposed to erect a mortuary tribute to one of the greatest names in the annals of Dentistry—"Dr. Chapin A. Harris." He was a native of New York State and lies buried in Mt. Olivet Cemetery near Baltimore—but his grave is in a condition of discreditable neglect.

The object of this appeal is to provide funds for a monumental tribute worthy of his name and to establish constructive evidence of the estimation in which he is held by the Dental Profession.

Mr. Ernest W. Keyser—the American sculptor now in Paris—has modelled from a photograph a remarkably faithful portrait bust of Dr. Harris and executed designs for the proposed monument.

Dr. Chapin A. Harris was born in 1806 at Pompey, Onandaga County, New York. He studied medicine, removed to Ohio and afterwards settled in Baltimore, Md., where he practiced dentistry until his death in 1860. He founded and assisted in the organization of the first dental college in the world—"The Baltimore College of Dental Surgery"—chartered in 1839. Was for many years its Professor of Dental Surgery. He edited the "American Journal of Dental Science," the first of its kind ever published—1839 until 1860—and was a voluminous contributor to other dental and medical journals. He was the author of "Principles and Practice of Dental Surgery." "Characteristics of the Human Teeth," "Diseases of the Maxillary Sinus," "Harris' Dental Dictionary," and edited "Fox's Natural History and Diseases of the Human Teeth," with additions, etc. These books are widely known to the civilized world.

Circulars soliciting contributions will be distributed over the entire country and it is believed that if each member of the profession co-operate by even the slightest subscription the aggregate result will determine whether the unquestioned services rendered by Prof. Harris to the Science of Dentistry should receive its meed of recognition and the tardy testimonial reach a successful consummation.

"The Snowden & Cowman Manufacturing Company" of Baltimore, one of the oldest in the United States and favorably known as reliable dealers in dental supplies have kindly consented to act as custodians to the "Harris Memorial Fund." Contributions are earnestly solicited and will be thankfully acknowledged. Address:

THE SNOWDEN & COWMAN MANFG. CO.,
No. 9 W. Fayette Street, Baltimore, Md.

